# Bacterial and Parasitic Assessment from Fingernails in Debre Markos, Northwest Ethiopia

**DOI:** 10.1155/2018/6532014

**Published:** 2018-10-18

**Authors:** Abeba Mengist, Yibeltal Aschale, Alemayehu Reta

**Affiliations:** Department of Medical Laboratory Science, College of Medical and Health Sciences, Debre Markos University, Debre Markos, Ethiopia

## Abstract

**Background:**

Food handlers with untrimmed finger nails could contribute or serve as a vehicle for the transmission of food poisoning pathogens.

**Objectives:**

This study was conducted to determine the prevalence of bacteria and intestinal parasites among food handlers and antibiotic susceptibility profile of the isolated bacteria in Debre Markos University, Ethiopia.

**Materials and Methods:**

This laboratory-based cross-sectional study involved 220 food handlers working in food service establishments in Debre Markos University between 1st January 2015 to 31th June 2016. Subjects' finger nail specimens of both hands were examined microscopically for intestinal parasites. For bacterial isolation, samples were cultured and bacterial species were identified following standard laboratory procedures. Antimicrobial susceptibility test was performed for all bacterial isolates by using Kirby-Bauer disk diffusion method.

**Results:**

Of the total 220 subjects examined, 29.5% showed positive culture for different bacterial species from their fingernail contents. Coagulase-negative Staphylococcus was the predominant bacteria species (12.3%) followed by *Staphylococcus aureus* (5%), *E. coli* (2.7%), *Klebsiella species* (2.7%), *Enterococcus species* (1.8%), *Pseudomonas aeruginosa* (1.8%), *Proteus species* (1.4%), *Citrobacter* species (1.4%), and *Serratia species* (0.9%). None of the food handlers showed positive culture for *Shigella* and salmonella and parasites in respect of their finger nail specimens. Isolation of bacteria in finger nail has significant association with finger nail status (*P*=0.044) and inverse relation with service years (*P*=0.048). All *Staphylococcus aureus* and coagulase-negative *Staphylococcus* species isolates were uniformly susceptible to vancomycin. Only one (9.1%) of *Staphylococcus aureus* isolates was resistant for methicillin.

**Conclusion:**

To prevent the food poisoning pathogens, implementation and adherence to infection are the key practices, specially food handlers with long finger nail harbor food debris, microbial contaminations, and allergens.

## 1. Background

Food-borne disease is a public health problem in developed and developing countries due to poor food handling and sanitation habit, inadequate food safety programs, lack of clean water supply, poverty, and lack of knowledge of food handlers [1]. According to the World Health Organization (WHO) statement, most of the populations suffer from food-borne diseases every year in both developed and developing countries [2]. The spread of food-borne disease through food handlers is a common and persistent problem worldwide [3]. Infected food handlers with poor hygiene practice working in food service establishments are potential sources and transmitters of the disease due to pathogenic organisms like infection with various intestinal helminths, protozoal, and enteropathogenic bacteria [4, 5]. They can transmit both enteric and nonenteric bacterial and parasitic infections through the food that they handled [3].

Microorganisms such as bacteria, parasites, and viruses are the common agents for food contamination. *Vibrio cholera, Campylobacter jejuni, Enterotoxigenic Escherichia coli, Salmonella typhi, Shigella species*, and *Polio* are the most common food-borne disease causing organisms in developing countries [6, 7]. Protozoan and helminthic parasites such as *Giardia lamblia*, *Entamoeba histolytica*, *Cryptosporidium species*, *Ascaris lumbricoides*, and *Enterobius vermicularis* are also important agents of food-borne disease. These infections in food handlers pose a significant threat to food consumers [6, 7].

Transmission of finger nail bacteria occurs through food, water, nails, and fingers contaminated with feces demonstrating the role of fecal-oral person-to-person transmission [8]. Food handlers who harbor and excrete bacteria may contaminate foods from their feces via their fingers and then to food preparation and servicing and lastly infect healthy individuals [9]. The area under the fingernail spreads pathogenic microorganisms via cross contamination, and it is challenging to clean when compared with other parts of the hand [5].

Food storage systems such as temperature and time, food preparation, handling, servicing practices, and food handlers' knowledge and skill are some of the factors that affect the safety of food directly or indirectly [10]. Biofilm formation is an important factor in persistence of microorganisms on the surface. Cells in a biofilm are embedded in an extracellular polymeric matrix constituent, proving resistant to conventional therapeutic doses of antimicrobial agents and clearance by the host response. Biofilm formation proceeds via initial adhesion to the surface and subsequent aggregation into multicellular structures. Thus, the development of a biofilm requires adhesive forces for the colonization of surfaces and cell interaction. Specifically, *S. epidermidis*is, one of the major biofilm-producing bacteria, works by attaching itself to several surfaces [11].

Various measures have been implemented to reduce incidence of food-borne diseases both in developed and developing countries. However, there has been increased occurrence of emerging and reemerging food-borne diseases. Among the factors responsible for this is the resistance of food-borne pathogens to antibiotics. Humans are exposed to resistant bacteria through sources such as food products, environment, and food handlers. Among the factors responsible for this occurrence and prevalence are poor food-production processes, inadequate food storage infrastructure, unhygienic food handling, limited resources, and poorly enforced regulatory standards [12].

Therefore, appropriate screening method is useful to detect bacterial and parasitic infections among food handler's finger nails, thus preventing probable illness and protecting the health of the consumers. Thus, this study was carried out to determine the prevalence and susceptibility pattern of finger nail bacteria and parasites among food handlers in Debre Markos University food service establishments to address appropriate recommendations for the enhancement of good food safety and sanitary conditions within food establishments in the University.

## 2. Materials and Methods

### 2.1. Study Design, Area, Period, and Participants

The present laboratory-based cross-sectional survey included 220 food handlers working in food establishments of Debre Markos University during the period from January 2015 to June 2016. The University is found in northwestern part of Ethiopia at Debre Markos town. The town is located 300 km northwest of Addis Ababa.

### 2.2. Data Collection

Data were collected by the data collectors after obtaining written informed consent using a well-structured questionnaire designed to obtain sociodemographic data and other relevant data related to food handlers' service year, status of medical screening, status of certification, education, and hand-hygiene practices from participants following their written informed consent and the ethical approval of the study from Debre Markos University ethics review board.

### 2.3. Sample Collection and Transport

Swab samples under the finger nails from both hands of each subject were collected using sterile-moistened cotton-tipped swabs and placed into a sterile test tube. Until inoculated on to respective cultured media, the samples were kept with normal saline in a test tube for not more than 5 minutes [13].

### 2.4. Culture and Identification

#### 2.4.1. Processing of Fingernail Swabs and Identification of Bacteria and Parasites

A single under finger nail swab obtained from each food handler was cultured immediately on Mannitol salt agar (MSA) for isolation of *S. aureus* and Coagulase-Negative *Staphylococci* (CNS). Finger nail swabs were cultured on to Salmonella-Shigella agar (Oxoid), MacConkey agar (Difco), and Blood agar (Oxoid) and then incubated at 37°C for 24 hours for isolation of Gram-negative bacteria. The bacterial colonies grown on the agar media were presumptively identified by colonial morphology and gram staining and a battery of biochemical tests like reaction on oxidase, catalase, simmon citrate, indole production, urease, motility, KIA, and gas and hydrogen sulfide (H_2_S) production [14]. For parasite identification, samples were examined microscopically following direct wet mount preparations in normal saline and iodine solution [10].

#### 2.4.2. Antimicrobial Susceptibility Testing

Antimicrobial susceptibility tests were performed on Muller Hinton Agar (Oxoid, Hampshire, UK) by disc diffusion method. The following antimicrobial agents were used for Gram-positive isolates: methicillin (10 *µ*g), penicillin (10 *µ*g), erythromycin (15 *µ*g), ampicillin (30 *µ*g), ciprofloxacin (10 *µ*g), tetracycline (30 *µ*g), cotrimoxazole (25 *µ*g), and vancomycin (30 *µ*g). To characterize Gram-negative isolates, ampicillin (10 *µ*g), tetracycline (30 *µ*g), chloramphenicol (30 *µ*g), gentamicin (10 *µ*g) and norfloxacillin (10 *µ*g) and cotrimoxazole (25 *µ*g) and ciprofloxacin (10 *µ*g) have been used. The susceptibility profiles (i.e., resistance and sensitivity) of the isolates were interpreted according to the National Committee for Clinical Laboratory Standards [15].

#### 2.4.3. Data Processing and Analysis

All statistical calculations were done using SPSS for windows version 20. Descriptive statistics were computed to determine the rate of *bacteria* and other variables. The relationships between the presence of bacteria and various risk factors were tested using the Chi square test. A *P* value of ≤0.05 was considered indicative of a statistically significant.

## 3. Result

### 3.1. Sociodemographic Data

Two hundred twenty food handlers were participated in this study. Among them, 69.1% were females and 30.9% were males. The age of the study participants ranged from 18 through 43 with a mean age of 25.1 (SD ± 4.1). Regarding their job, 45.9% had one to two years of work experience and only 30.9% had more than two years of work experience (Table 1).

In this study, the majority (97.3%) and only few (17%) of food handlers had a habit of hand washing after toilet and after touching different body parts, respectively (Table 2).

### 3.2. Prevalence of Bacteria Isolated from Finger Nail of Food Handlers

The frequency and type of bacteria isolated from *fingernail content* of the 220 food handlers studied are presented in Table 3. Bacteria isolated include coagulase-negative Staphylococcus (12.3%), *Staphylococcus aureus* (5%), *Escherichia coli* (2.7%), *Klebsiella* species (2.7%), *Enterococcus* species (1.8%), *Pseudomonas aeruginosa* (1.8%), *Proteus* species (1.4%), *Citrobacter* species (1.4%), and *Serratia* species (0.9%). While no bacteria were isolated from the finger nail content of 70.5% of participants. None of the food handlers showed positive culture for Shigella and salmonella in respect of their finger nail specimens. No more than one enteric bacterium was observed in the subject under study. In addition, no intestinal parasites were detected from the samples of fingernail contents.

In this study, different factors were assessed for possible association with finger nail bacterial isolation rate among the study participants (Table 2 and 4). The number of positive cultures from finger nail contents was higher among female subjects (30.7%) than those of male subjects (26.9%), but the difference was not statistically significant (*P*=0.564) (Table 4).

The isolation rate of bacteria in finger nail of food handlers was relatively higher 22(43.1%) among food handlers served for a period of less than one year and lower 16(23.5%)s among those served for a period of greater than 2 years (Table 4). Therefore, the inverse relationship between service year and finger nail bacterial isolation rate was statistically significant (*P*=0.048). In addition, food handlers with long finger nails showed more 33(37.1%)s bacterial isolation rate with their finger nails as compared to those food handlers with short (properly cut) finger nails 32(24.4%) (*P*=0.044) (Table 2). However, the other expected risk factors (i.e., age, educational background, medical check-up, food hygiene training, and hand washing habit) had not been found to be associated with bacterial fingernail rate (Table 2 and 4).

### 3.3. Antimicrobial Susceptibility Pattern of Isolated Pathogens

All *Staphylococcus aureus* and coagulase-negative *Staphylococcus* species isolates were uniformly susceptible to vancomycin. Relatively, *Staphylococcus aureus* showed low resistance to methicillin (9.1%), cirofloxacin (9.1%) and erythromycin (18.2%), and cotrimoxazole (18.2%); high resistance to penicillin (63.6%) and ampicillin (63.6%) followed by amoxycillin and tetracycline with 54.5%% and 45.5%, respectively (Table 5). The susceptibility profile of the Gram-negative isolates is presented in Table 6.

## 4. Discussion

Improper food handlings practices by food handlers may cause food contamination and food-borne diseases, which may pose a possible risk to community or customers [16]. Therefore, this study was undertaken to assess the prevalence of bacteria and intestinal parasites among food handlers and antibiotic susceptibility profile of the isolated bacteria in Debre Markos University, Ethiopia.

In this study of 220 food handlers examined, 29.5% were carriers of enteric bacteria including coagulase-negative Staphylococcus (12.3%), *Staphylococcus aureus* (5%), *Escherichia coli* (2.7%), *Klebsiella* species (2.7%), *Enterococcus* species (1.8%), *Pseudomonas aeruginosa* (1.8%), *Proteus* species (1.4%), Citrobacter species (1.4%), and *Serratia* species (0.9%) in their finger nail. Therefore, food handlers should ensure that their finger nails are trimmed. Similar types of bacterial isolate were identified among food handlers in other parts of Ethiopia including Jimma and Gondar [10, 17]. Our finding also goes parallel with different studies carried out in other countries like Nigeria [18], Iran [19], Brazil [20], and Turkey [21].

In the present study, approximately one-sixth of cultures of fingernail contents was found to be positive for coagulase-negative Staphylococci (12.3%) followed by *Staphylococcus aureus* (5%). Our results are in agreement with previous studies reported from other parts of the country [10, 16, 17] and are similar to findings of Zaglool et al. from Saudi Arabia who reported these bacteria as the most common pathogen isolated from food handlers [9]. Coagulase-negative Staphylococci are the normal flora of the skin, and this is the reason why high prevalence in this study. In addition, isolation of *Staphylococcus aureus* in this study was because it is the true pathogenic bacteria included in the resident microflora of the skin and nose. Food handlers can easily contaminate food with *Staphylococcus aureus* (common cause of food poising) if they do not wash their hands properly after using toilet and after making contact with their nose [22]. Tambekar et al. also reported the reduction in the number of pathogens after hand washing [23].

Different species of Enterobacteriaceae were isolated in 11.5% of food handlers (data not shown) in the present study. *Klebsiella* and *Escherichia coli* were the predominates, supporting the concept of contamination by fecal bacteria due to inadequate handwashing by the food handlers, which are a cause of concern for the public [24]. Furthermore, only 15.5% of the food handlers in our institution had been trained in safe food handling practices.

*Escherichia coli* is a normal flora usually present in the intestine even though some serotypes (i.e., 0157 : H7) can cause serious diseases to humans [25]. It is normally absent in hands, and the presence of this bacterium gives a clue of current fecal contamination with enteric pathogens [26]. Foods that are contaminated with *Escherichia coli* and *Staphylococcus aureus* that do not require further heat treatment could cause food-borne illnesses [27]. *Escherichia coli* was detected on the hands of 2.7% of food handlers' in the current study, which is in accordance with 1.8%–3.9% isolation rates reported in earlier studies [17, 28, 29]. However, this figure is lower than 22%, 10.9%, and 7.8% carriage reported in Jimma, Iran, and Turkey, respectively [16, 19, 28]. The difference between our results and the other studies may be attributed to sampling techniques as well as the different used methods for detection.

Pathogens that can be originated from undercooked or uncooked animal products like *Proteus* and *Klebsiella* can contaminate hands of food handlers from where they could be transferred to foods and the customers [16]. *Pseudomonas aeruginosa* is resistant to most antibiotics and disinfectants; hence, when transferred to foods through the nails of food handlers, food poisoning may occur, and isolating or identifying this pathogen is dangerous [27].

In the present study, no intestinal parasites were detected in the fingernails of food handlers. This finding is in line with the result obtained from study done earlier in Gondar town, Ethiopia and Makkah, Saudi Arabia [9, 10]. Though, other previous reports indicated the presence of intestinal parasites in the fingernails contents of study participants [29, 30]. Likewise, all of the food handlers were not positive for salmonella and *Shigella* species in respect of their fingernail contents in the present study, which is also in line with previous studies done in Gondar [10, 17] and Abuja, Nigeria [29].

The *Staphylococcus aureus* and coagulase-negative Staphylococcus found in the finger nail content were resistant to multiple antibiotics in this study. *Staphylococcus aureus* isolated from finger nail contents was resistant to methicillin. If it is transmitted to patients, it may cause epidemics in patients. The finding of this study is consistent with the previous study done in Gondar University Cafeteria, Northwest Ethiopia [17].

In the current study, there was significant association between bacterial isolation rate and service years (*P*=0.048). This finding is in line with the result obtained from the previous study done in Debre Markos Ethiopia [13]. However, this was in contrary with the findings from Addis Ababa and Arba Minch University, South Ethiopia [31, 32] where no statistical significant association between bacterial isolation and service was seen. This result indicated that food handlers with more work experience have less risk of bacterial finger nail isolation. This could be explained as food handlers with more work experience have better personal hygienic practices than inexperienced food handlers [13].

Colonization of bacteria on hands can be facilitated by having untrimmed fingernails because it makes hand washing difficult and less effective. Wachukwu et al. have showed that food handlers with long nail become colonized and become a possible risk for transmission of pathogens [27]. In addition, a study conducted by Lau et al. on removal of *Escherichia coli* hands with natural or artificial fingernails indicated that untrimmed fingernails tend to carries more microorganisms than untrimmed nails [33]. Our study also indicated statistically significant association between the isolation of bacteria and finger nail status (*P*=0.044).

In our study, no significant association was found for finger nail bacterial content by sex, age, educational background, medical checkup, training status, and hand washing habit of food handlers. However previous study conducted in Jimma indicates significant association between bacterial hand contamination rates with age [16]. Similarly, finding in Sari, northern Iran, showed that statistical significant association was observed particularly bacterial infestation comparable for educational level and handwashing practice after using toilet [22]. But this may be due to the small sample size.

## 5. Conclusion

In general, the present study emphasized the use of different intervention measures that can be used to decrease or eliminate contamination of foods by food-handlers as well as spread of pathogens to the customers or the public. Therefore, creating awareness specially on food handling practices and hygienic measures of food handlers is a crucial issue to prevent the food poisoning pathogens. Specially, individuals with long finger nail harbor food debris, microbial contaminations, and allergens. Therefore, their use should be under control or supervision by the responsible body in the institution with much customers.

## Figures and Tables

**Table 1:** Sociodemographic characteristics of food handlers (*n*=220) working at food service establishments in Debre Markos University (1st January 2015 to 31st June 2016).

Sociodemographic characteristics	Frequency	Percent (%)
*Sex*		
Male	67	30.5
Female	153	69.5

*Age in years*		
≤20	20	9.1
21–30	189	85.9
31–40	6	2.7
41–50	5	2.3

*Educational level*		
Primary (1–8)	64	29.1
Secondary (9–12)	71	32.3
Postsecondary(>12)	85	38.6

*Service* years		
<1	51	23.2
1-2	101	45.9
>2	68	30.9

*Certified in food preparation and handling*		
Yes	34	15.5
No	186	84.5

*Medical check-up*		
Yes	138	62.7
No	82	37.3

**Table 2:** Hygienic practices of food handlers (*n*=220), in relation to finger nail bacterial isolates, working at food service establishments in Debre Markos University (1st January 2015 to 31st June 2016).

Variables	Total	Bacterial culture result from finger nail		Association
		Positive *n* (%)	Negative *n* (%)	
*Finger nail status*				
Trimmed	131	32 (24.4)	99 (75.6)	*X* ^2^=4.075
Not trimmed	89	33 (37.1)	56 (62.9)	*P*=0.044

*Hand washing after using the toilet*				
Yes	214	63 (29.4)	151 (70.6)	*X* ^2^=0.043
No	6	2 (33.3)	4 (66.7)	*P*=0.837

*Hand washing after* touching *body parts*				
Yes	22	5 (22.7)	17 (77.3)	*X* ^2^=0.546
No	198	60 (30.3)	138 (69.7)	*P*=0.460

**Table 3:** Types of bacteria isolated from finger nail content of food handlers (*n*=220) working at food service establishments in Debre Markos University (1st January 2015 to 31st June 2016).

Bacteria	Frequency	Percent (%)
*Coag. Neg. Staph*	27	12.3
*S. aureus*	11	5
*Klebsiella* spp.	6	2.7
*Escherichia coli*	6	2.7
*Enterococcus* spp.	4	1.8
*Proteus species*	3	1.4
*Pseudomonas aeruginosa*	3	1.4
*Serratia species*	2	0.9
*Citrobacter species*	3	1.4
None	155	70.5
**Total**	**220**	**100**

Coag. Neg. Staph: coagulase-negative Staphylococcus.

**Table 4:** Sociodemographic characteristics of food handlers (*n*=220), in relation to finger nail bacterial positivity, working at food service establishments in Debre Markos University (1st January 2015 to 31st June 2016).

Variables	Total	Bacterial culture result from finger nail		Association
		Positive *n* (%)	Negative *n* (%)	
*Sex*				
Male	67	18 (26.9)	49 (73.1)	*X* ^2^=0.332
Female	153	47 (30.7)	106 (69.3)	*P*=0.564

*Age in years*				
≤20	20	6 (30)	14 (70)	
21–30	189	56 (29.6)	133 (70.4)	
31–40	6	1 (16.7)	5 (83.3)	*X* ^2^=0.743
41–50	5	2 (40)	3 (60)	*P*=0.863

*Educational level*				
Primary (1–8)	64	20 (31.2)	44 (68.8)	
Secondary (9–12)	71	20 (28.2)	51 (71.8)	*X* ^2^=0.155
Postsecondary(>12)	85	25 (29.4)	60 (70.6)	*P*=0.926

*Service* years				
<1	51	22 (43.1)	29 (56.9)	*X* ^2^=6.092
1–2	101	27 (26.7)	74 (73.3)	*P*=0.048
>2	68	16 (23.5)	52 (76.5)	

*Certified in food preparation and handling*				
Yes	34	10 (29.4)	24 (70.6%)	*X* ^2^=0.000
No	186	55 (29.6)	131 (70.4)	*P*=0.985

*Medical check-up*				
Yes	138	43 (31.2)	95 (68.8)	*X* ^2^=0.463
No	82	22 (26.8)	60 (73.2)	*P*=0.496

**Table 5:** Antimicrobial resistance pattern of *S. aureus* and CNS isolated from finger nail cultures of food handlers working at food service establishments in Debre Markos University (1st January 2015 to 31st June 2016).

Antimicrobial agents tested	Sensitivity pattern	*Staphylococcus aureus* (*n*=11), no. (%)	CNS (*n*=27), no. (%)
Methicillin	S	10 (90.9)	25 (92.6)
	R	1 (9.1)	2 (7.4)

Vancomycin	S	11 (100)	27 (100)
	R	0 (0)	0 (0)

Amoxicillin	S	5 (45.5)	17 (63)
	R	6 (54.5)	10 (37)

Ampicillin	S	4 (36.4)	19 (70.4)
	R	7 (63.6)	8 (29.6)

Penicillin	S	4 (36.4)	16 (52.3)
	R	7 (63.6)	11 (40.7)

Ciprofloxacin	S	10 (90.9)	22 (81.5)
	R	1 (9.1)	5 (18.5)

Tetracycline	S	5 (45.5)	15 (55.6)
	R	6 (54.5)	12 (44.44)

Erythromycin	S	9 (81.8)	24 (88.9)
	R	2 (18.2)	3 (11.1)

Cotrimoxazole	S	9 (81.8)	25 (92.6)
	R	2 (18.2)	2 (7.4)

CNS: coagulase-negative Staphylococcus aureus, N = number, R = resistant, S = sensitive.

**Table 6:** Antimicrobial resistance pattern of different Gram-negative bacterial species isolated from finger nail content of food handlers working at food service establishments in Debre Markos University (1st January 2015 to 31st June 2016).

Bacterial isolate	Total	Sensitivity pattern n (%)	Ampicillin	Gentam	Tetracy	Ciprof	Cotrimoxazol	Chlora	Norflo
*Escherichia coli*	6	S	2 (33.3)	4 (66.7)	3 (50)	6 (100)	5 (83.3)	3 (50)	6 (100)
		R	4 (66.7)	2 (33.3)	3 (50)	0 (0)	1 (16.7)	3 (50)	0 (0)

*Klebsiella spp.*	6	S	2 (33.3)	4 (66.7)	4 (66.7)	6 (100)	5 (83.3)	4 (66.7)	6 (100)
		R	4 (66.7)	2 (33.3)	2 (33.3)	0 (0)	1 (16.7)	2 (33.3)	0 (0)

*Citrobacter spp*	3	S	2 (66.7)	1 (33.3)	2 (66.7)	3 (100)	3 (100)	1 (33.3)	3 (100)
		R	1 (33.3)	2 (66.7)	3 (33)	0 (0)	0 (0)	2 (66.7)	0 (0)

*Enterobacter spp*	4	S	3 (75)	3 (75)	3 (75)	4 (100)	4 (100)	3 (75)	4 (100)
		R	1 (25)	1 (25)	1 (25)	0 (0)		1 (25)	0 (0)

*Pseudomonas aeruginosa*	3	S	1 (33.3)	2 (66.7)	2 (66.7)	3 (100)	2 (66.7)	2 (66.7)	3 (100)
		R	2 (66.7)	1 (33.3)	1 (33.3)	0 (0)	1 (33.3)	1 (33.3)	0 (0)

*Seratia*	2	S	1 (50)	1 (50)	2 (100)	2 (100)	2 (100)	1 (50)	2 (100)
		R	1 (50)	1 (50)	0 (0)	0 (0)	0 (0)	1 (50)	0 (0)

*Proteus*	3	S	2 (66.7)	3 (100)	2 (66.7)	3 (100)	3 (100)	1 (33.3)	3 (100)
		R	1 (33.3)	0 (0)	1 (33)	0 (0)	0 (0)	2 (66.7)	0 (0)

SPP: species; Gentam: gentamycin; Tetracy: tetracycline; Ciprof: ciprofloxacin.

## Data Availability

The data used to support the findings of this study are available from the corresponding author upon request.

## Abbreviations

WHO: World Health Organization.
